# Supplementary figures and images for: Analyses of emerging macrocyclic lactone resistance: Speed and signature of ivermectin and moxidectin selection and evidence of a shared genetic locus
Source: PLoS Pathog. 2025 Oct 6;21(10):e1013578. doi: 10.1371/journal.ppat.1013578 (PMC12517496; doi:10.1371/journal.ppat.1013578)

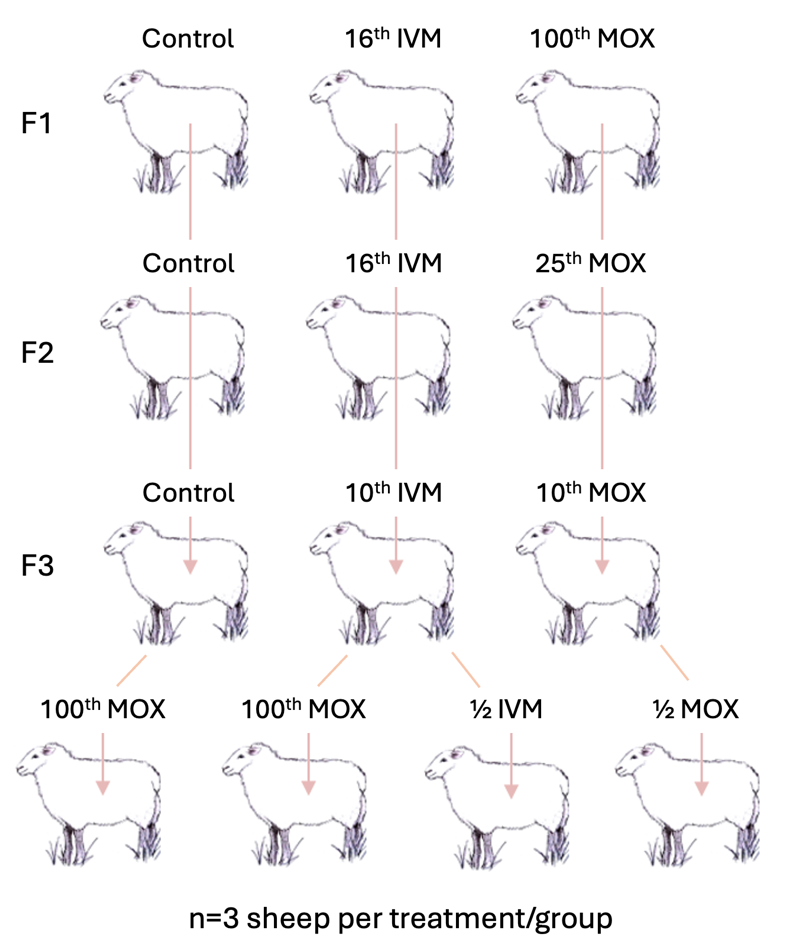


**S1 Fig**

Supplement: S1 Fig — (DOCX) [file ppat.1013578.s001.docx]

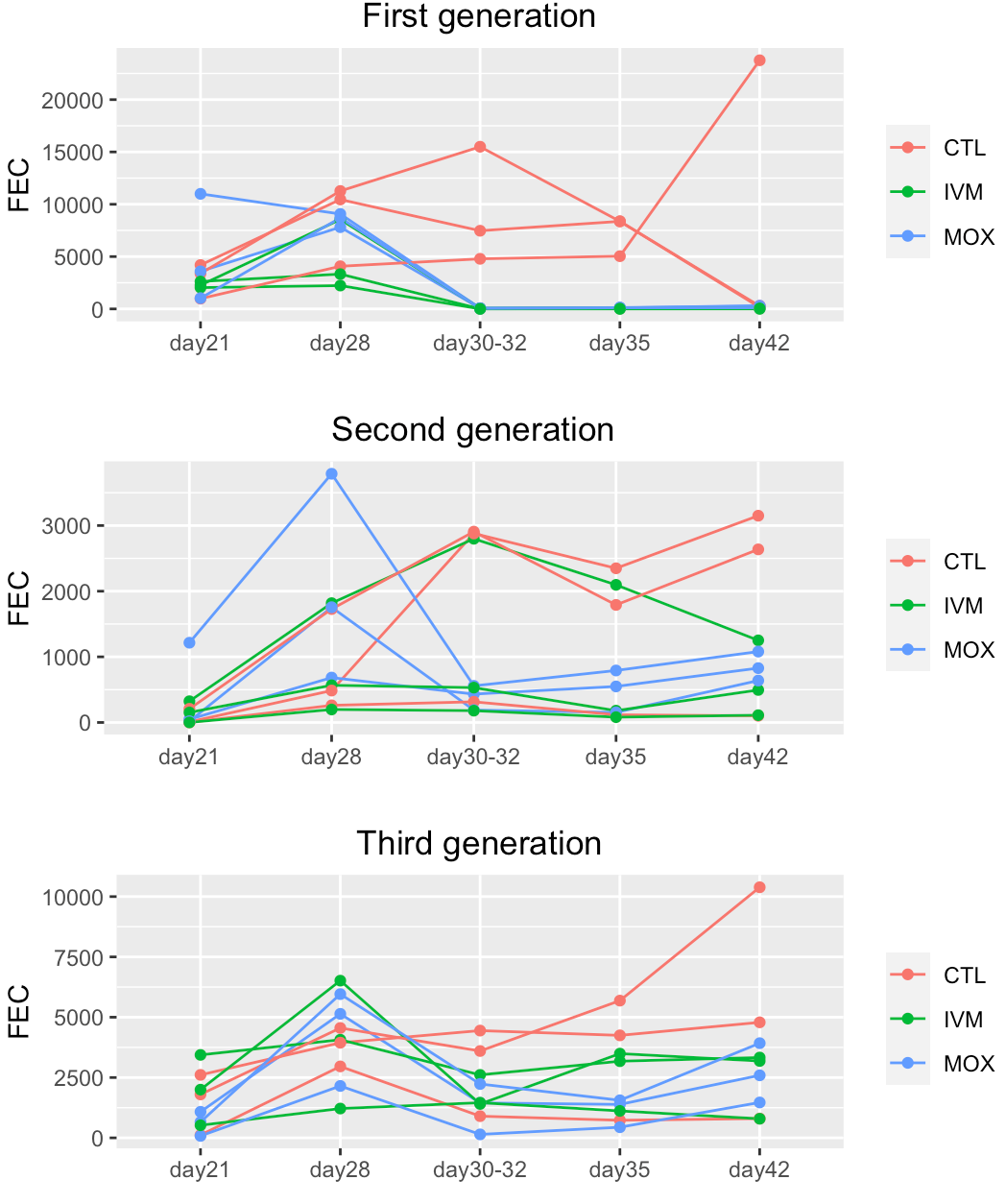


**S2 Fig.**

Supplement: S2 Fig — Treatment was on day 28 post infection. (DOCX) [file ppat.1013578.s002.docx]

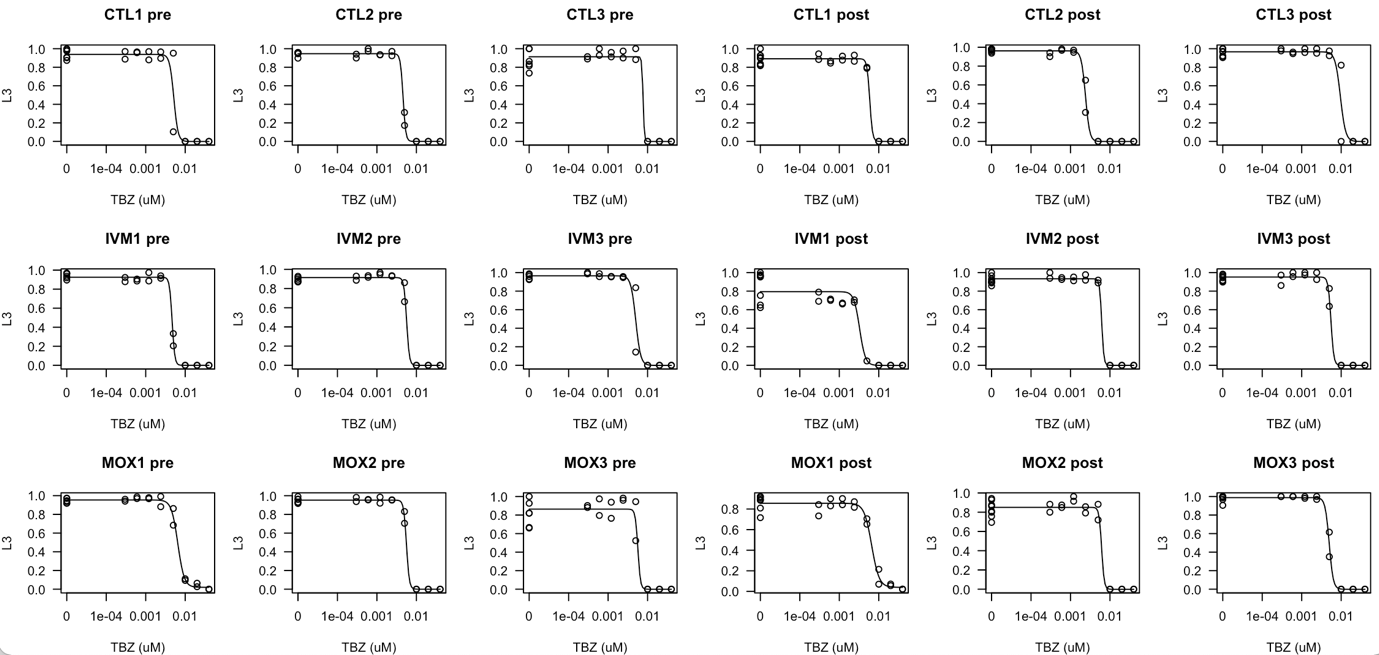


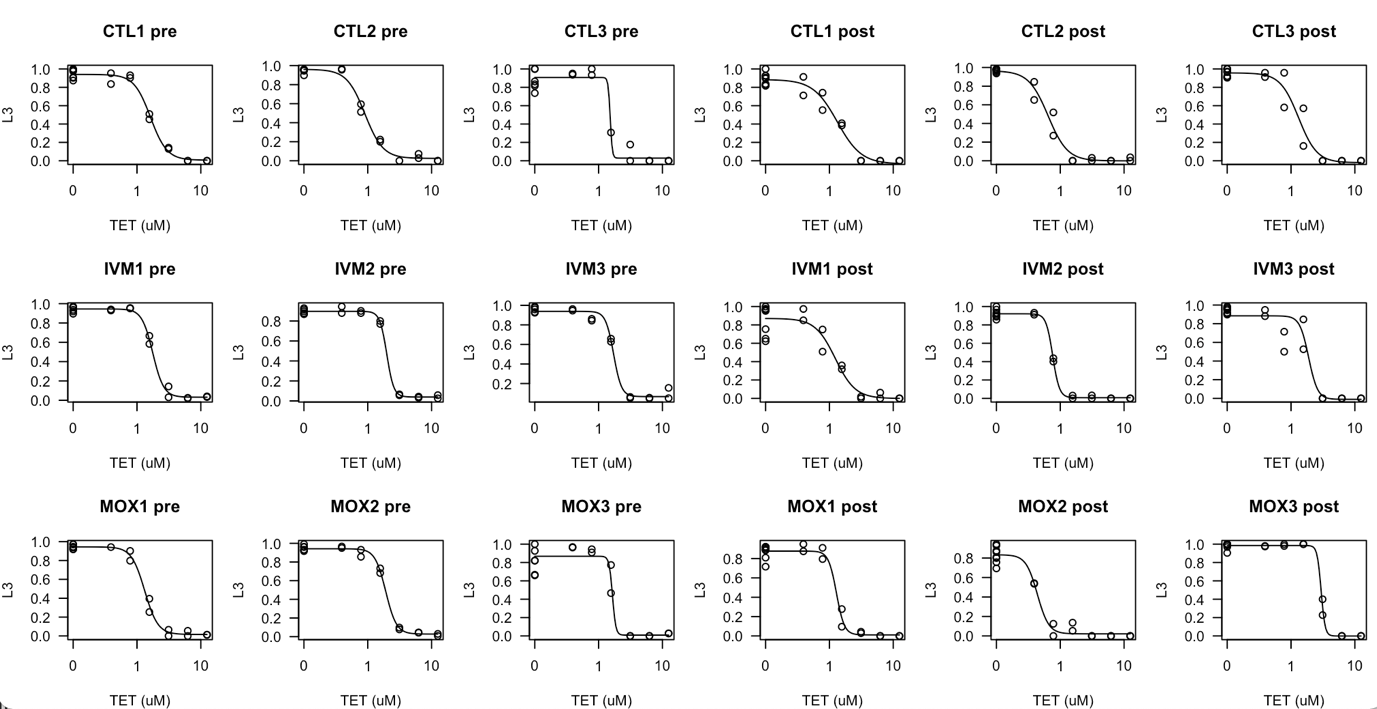


**S3 Fig.**

Supplement: S3 Fig — Figure titles refer to the drug-selected lines that were subjected to the assays. (DOCX) [file ppat.1013578.s003.docx]

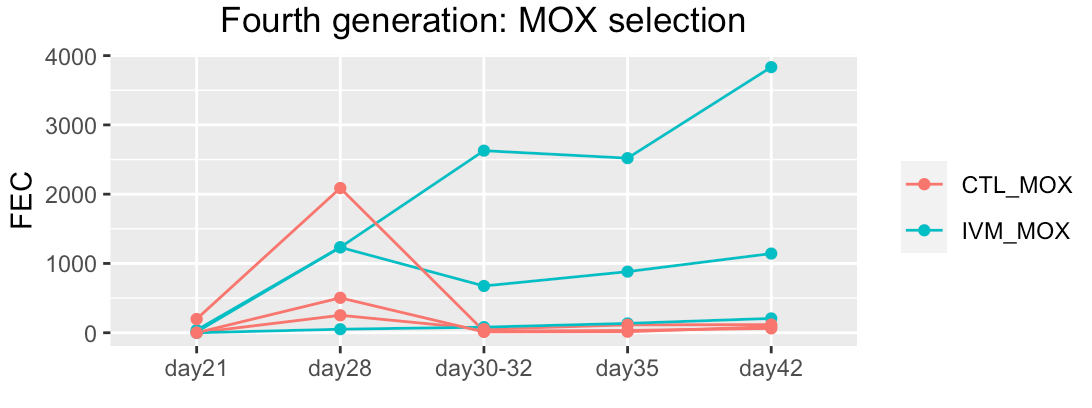


**S4 Fig.**

Supplement: S4 Fig — Treatment was on day 28 post infection. (DOCX) [file ppat.1013578.s004.docx]

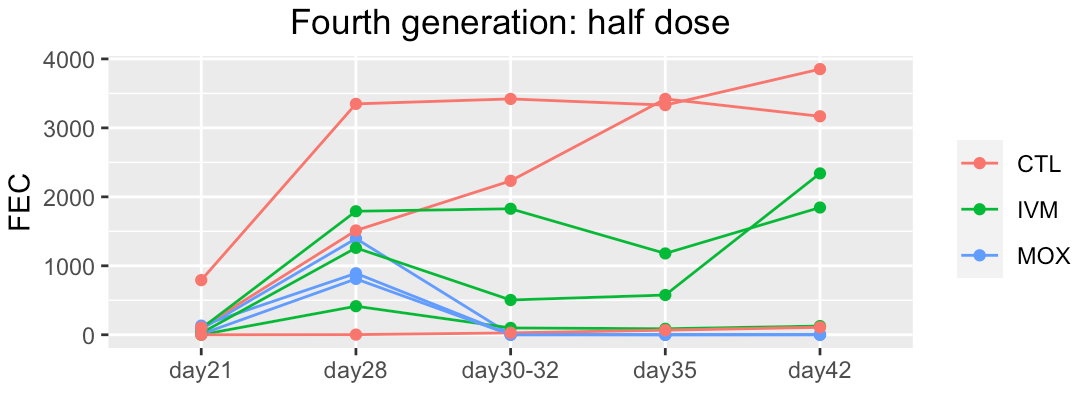


**S5 Fig.**

Supplement: S5 Fig — Treatment was on day 28 post infection. (DOCX) [file ppat.1013578.s005.docx]

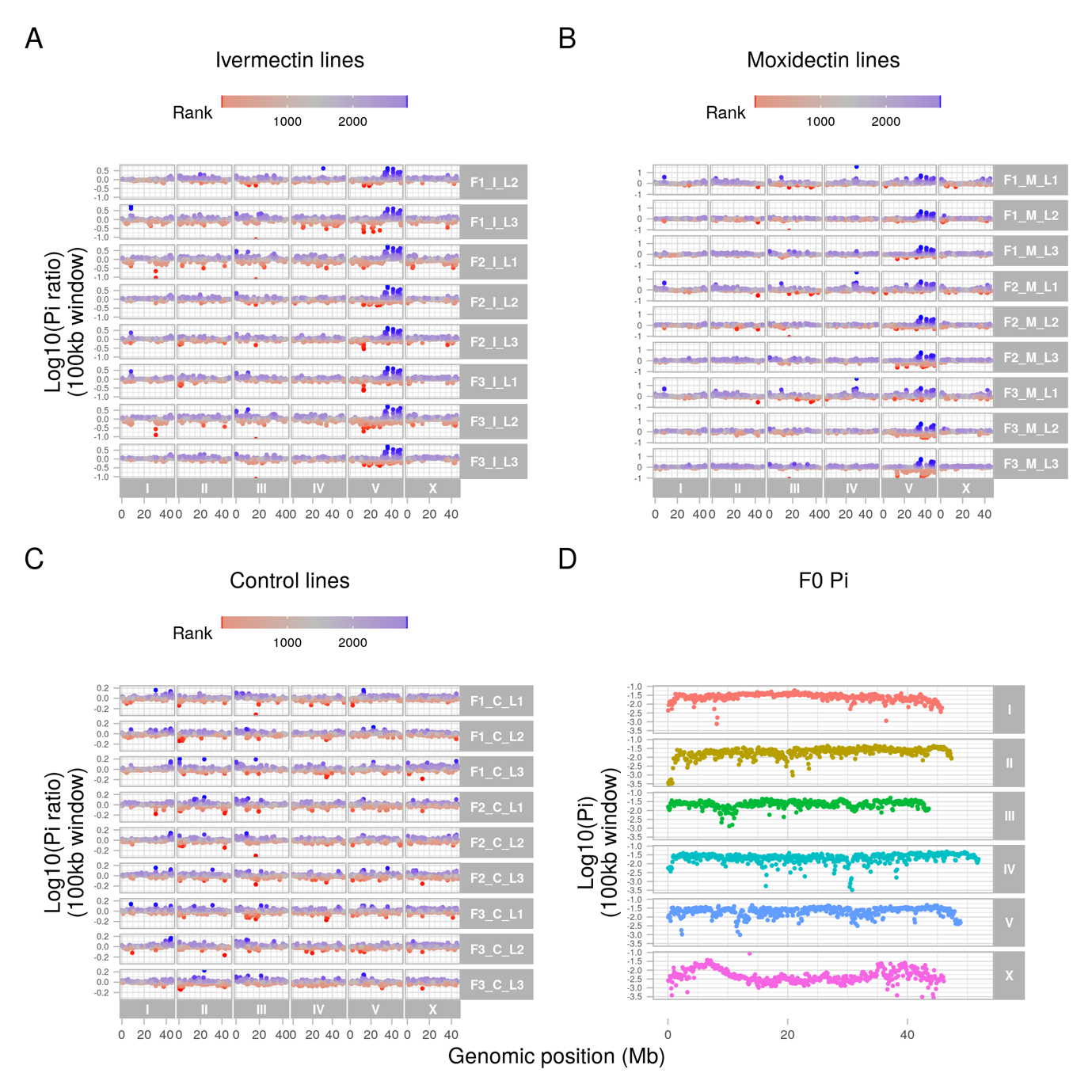


**S6 Fig.**

Supplement: S6 Fig — (A-C) Ratio of nucleotide diversity in three generations (F1 to F3) of sub-therapeutic selection in comparison to F0 sample population. Key: C = control, I = ivermectin, M = moxidectin. L1 to L3 indicate biological replicate selection lines. Pi ratio = Normalised Sample θπ/ Normalised F0 θπ. Nucleotide diversity for each sample window was normalised by dividing by the genome-wide median diversity. Colour indicates the rank within the treatment group, where blue indicates a higher diversity than F0, and red a lower diversity. Note that the rank indicates the genome-wide ranking. No sequencing data was available for F1_I_L1. (D) Nucleotide diversity (θπ) of the F0 sample along all chromosomes. More negative values indicate lower diversity. (DOCX) [file ppat.1013578.s006.docx]

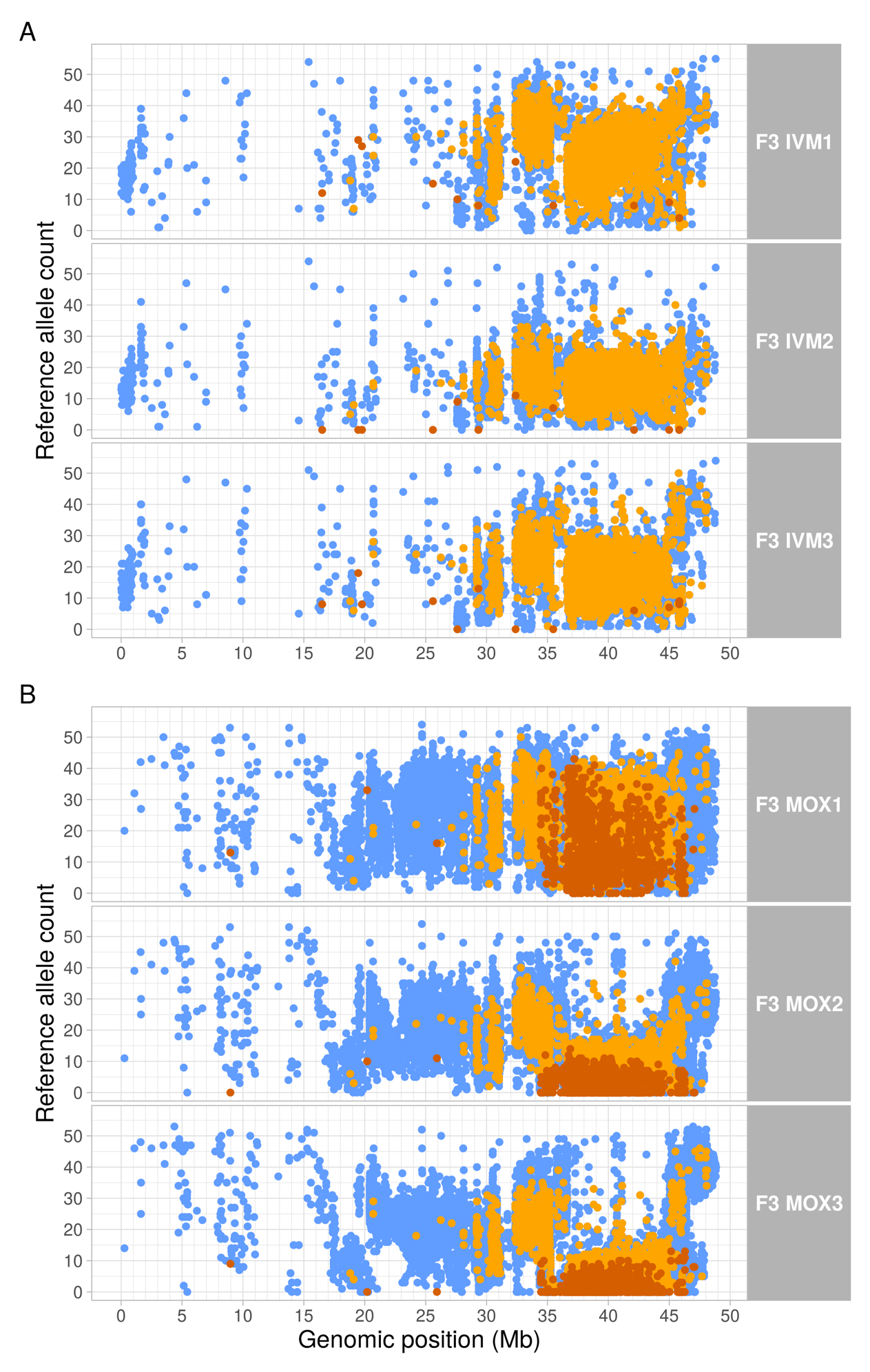


**S7 Fig.**

Supplement: S7 Fig — Using a 57X subsampled input file, SNPs were identified which reduced in the reference allele count from the F0 to the F3 generation (F0 > F1 > F2 > F3) in selected lines, but did not do so in control lines. In addition, SNPs were identified where the reference allele was zero in any F3 selected sample, but >20 in the F0 sample and F3 control samples. (A) Ivermectin selected F3 samples, (B) Moxidectin selected F3 samples. Key to colours: Blue - reference allele reduces in all selected lines of either ivermectin (IVM1 to IVM3) OR moxidectin (MOX1 to MOX3) from F0 to F3, Orange - reference allele reduces in all ivermectin AND moxidectin lines from F0 to F3, Red - reference allele count is zero in at least one F3 sample of the ivermectin (A) OR moxidectin (B) lines but it is at least 20 in the F0 and F3 control line samples. (DOCX) [file ppat.1013578.s007.docx]

**
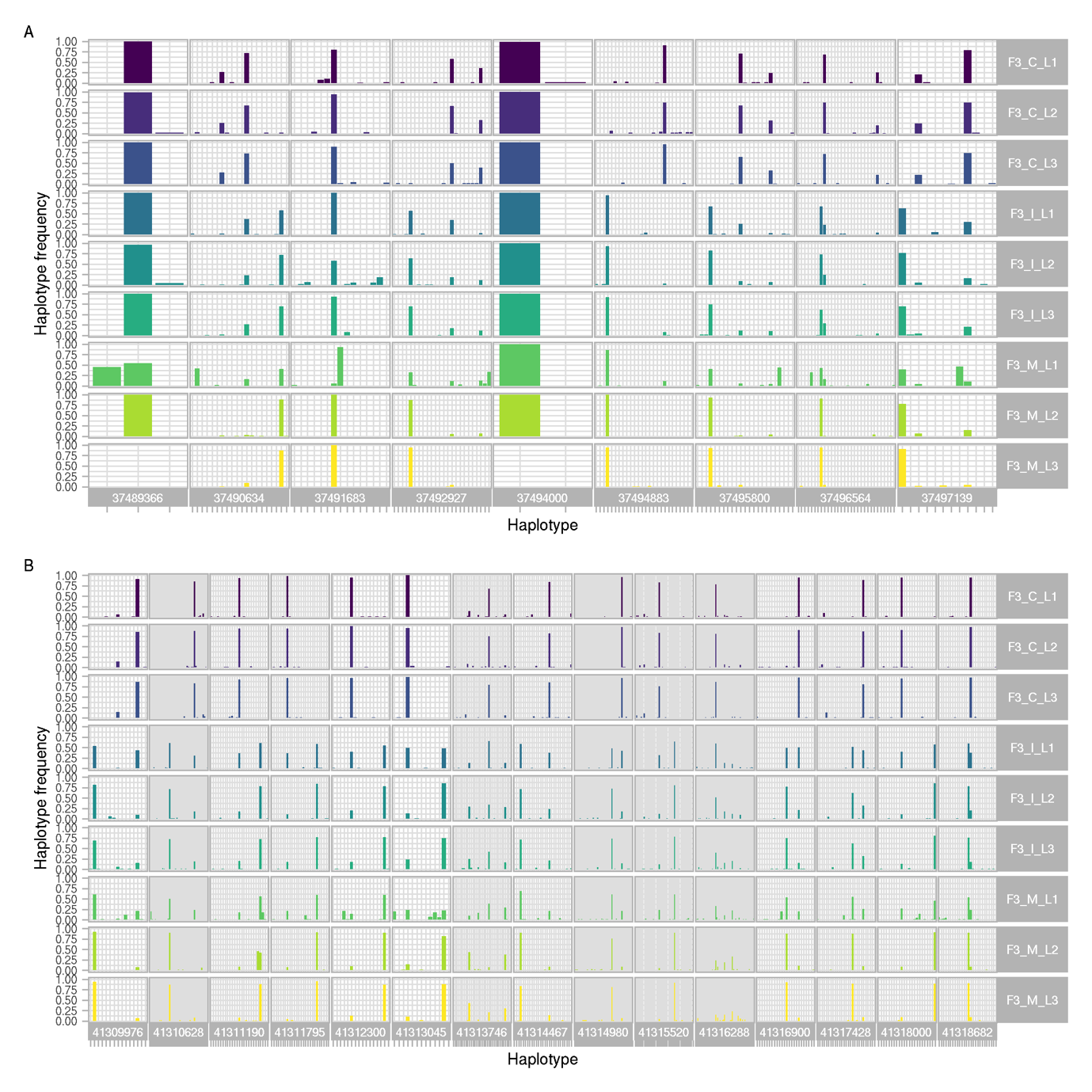
**

**S8 Fig.**

Supplement: S8 Fig — (A) HCON_00155390 (Hc-cky-1), Chr V:37,487,982–37,497,398 (B) HCON_00158050 (a serpentine receptor, class E), Chr V:41,309,214–41,318,844. Colours denote samples. Haplotypes are spread along the x-axis, with the mid position of each microhaplotype (based on start and end SNP) provided. Only haplotype frequencies >0.01 are shown. (DOCX) [file ppat.1013578.s008.docx]

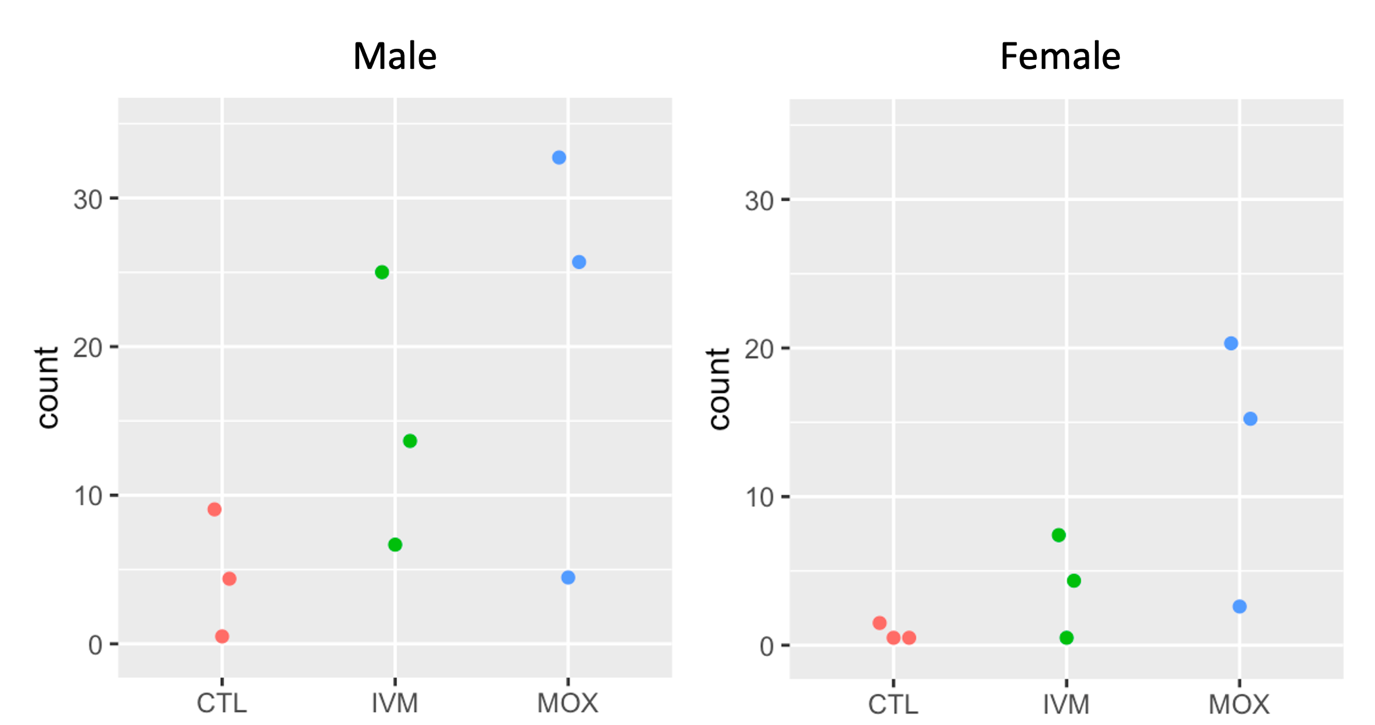


**S9 Fig.**

Supplement: S9 Fig — Read counts were normalised by size factor as per the DESEQ2 model. (DOCX) [file ppat.1013578.s009.docx]
