# Supplementary material for: Analyses of emerging macrocyclic lactone resistance: Speed and signature of ivermectin and moxidectin selection and evidence of a shared genetic locus
Source: PLoS Pathog. 2025 Oct 6;21(10):e1013578. doi: 10.1371/journal.ppat.1013578 (PMC12517496; doi:10.1371/journal.ppat.1013578)
Supplement: Attachment — Submitted filename: PLOS Path Response to Reviewers 18.09.25.docx [file ppat.1013578.s015.docx]

Dear Professor Martin,

Thank you very much for the reviewers’ comments on our manuscript and for the invitation to submit a revised version to PLOS Pathogens. We thank all three reviewers for their feedback and comments, which we found to be positive and constructive, and we believe these revisions have improved the manuscript.

Please find our responses to each point detailed below (in italics).

Thank you again for considering our submission.

Your sincerely,

Jennifer McIntyre and Roz Laing

**Part I - Summary**

Reviewer #1: This manuscript explores the relationship between resistance to two macrocyclic lactone anthelmintics, ivermectin and moxidectin. Although moxidectin’s increased efficacy against ivermectin-resistant animals has been well documented, the current work explores how selection for resistance to each drug, caused by underdosing, affects the efficacy of both drugs against a population. Generally, I believe the work is well done by a group of authors who have continued to push our understanding of ML resistance in Haemonchus. However, I have one major comment and multiple smaller comments that should be addressed for more detail and clarity to the information being presented.

Reviewer #2: This study reports the first body of work that explores the development of anthelmintic resistance 'in real time' and combines well designed experimental infections with complex genome-wide analyses. As such it represents a significant contribution to the field. With a focus on the macrocyclic lactones, ivermectin and moxidectin, its relevance extends beyond the veterinary sector to encompass human health. It is a valuable study that highlights the detrimental impact of sub-therapeutic doses of anthelmintics.

Reviewer #3: In McIntyre et al, the authors demonstrate that even single, subtherapeutic doses of ivermectin or moxidectin rapidly select for preexisting resistance alleles in Haemonchus contortus, pinpointing a shared hotspot on chromosome V. By subjecting a drug susceptible isolate to successive low dose treatments, they show that three rounds of underdosing with ivermectin are sufficient to confer full clinical resistance, and cross resistance to moxidectin, whereas moxidectin underdosing produces a strong genomic signature without equivalent phenotypic protection. These findings provide the first direct evidence that underdosing can swiftly drive anthelmintic resistance and identify a genetic marker for early surveillance and intervention. The manuscript is well written and appears sound, and the main conclusions are well supported. Before acceptance the following comments/concerns need to be addressed.

1. RNA-seq analysis. Post-treatment adult samples were used in the RNA-seq analysis. It is unclear when these adult worms were collected. Were they collected 14 days after treatment? Several genes were found to be downregulated in the selected lines (Fig. 7 and Table 6). Given the high SNP density, which is likely to be even higher in the selected lines at the selected locus compared to controls, RNA-seq reads may have been too divergent from the reference genome for the aligner to map them accurately. This could result in lower read counts. HISAT2, when used with default settings, cannot accommodate a large number of mismatches per read. Based on our experience, STAR performs better than HISAT2 for aligning divergent reads. STAR also allows adjustment of mismatch tolerance (--outFilterMismatchNmax). Only a small number of genes were differentially expressed across all four pairwise comparisons (Table 6). However, no functional descriptions are provided for these genes. Do their predicted functions offer any insight into possible mechanisms of resistance?

***RESPONSE:*** *Thank you for these comments on the RNA-seq analysis. For the age of adult worms please see line 142 in Materials and Methods ‘Adult worms were harvested at post-mortem on day 42 post-infection (14 days post-treatment)’. Thank you for recommending the use of STAR to align reads to the genome rather than HISAT2 – this was very helpful! We re-ran the analysis with STAR and, as the reviewer correctly predicted, were able to map a greater proportion of divergent reads (9.9M more reads on average, of which 4.5M were mapped to Chromosome V). The results and discussion have been updated with this new analysis (Section 6). None of the differentially expressed genes have predicted functions that link them to a resistance mechanism as far as we can determine, other than our speculations about cky-1; some genes have no functional annotation, and for those that do, predictions based on homology with C. elegans and/or conserved protein domains may not reflect their true function in Haemonchus and/or they may contribute to a novel mechanism of resistance that is not expected. A list of C. elegans homologues and Interpro domains for the differentially expressed genes have been added as Supplementary Table 5.*

2. Analysis of longitudinal allele frequency change (Fig. 6 and Supplementary Fig. 6). This analysis implicitly assumes that the reference allele represents the susceptible allele. While this may be true for most SNPs, it might not hold for all cases. It would be useful to explicitly confirm whether the major allele in the F0 population corresponds to the reference allele. If this assumption does not hold for a substantial number of SNPs, the analysis could be revised using the major allele in F0 rather than using the reference allele.

***RESPONSE:*** *Thank you for your comment. You are quite correct that we tend to think of the reference allele as ‘susceptible’ and that this may not be the case for all SNPs. Of the SNPs used for the allele frequency model (~ 1 million), 86.9% had the reference allele as the major allele in the F0 sample. However, neither analysis relies on the reference allele being sensitive to MLs – both focus on the change in the reference allele from F0 to F3 and compare selected lines with control lines. While Supplementary Figure 6 only plots data where the reference allele is reducing, thus excluding cases where the reference allele could be resistant, or present in a resistant haplotype under selection, the allele frequency model considers both scenarios. We have tried to make this clearer in the results: we have changed ‘reduction’ to ‘change’ which is more accurate and less biased. We have also highlighted that some of the reference SNPs that increased in selected lines were statistically significant and have added the following sentence:*

*Results and Discussion Section 5: “This could mean that one or more of these reference alleles are resistant, or more likely that these represent alternative sensitive alleles segregating within the Haemonchus population.”*

*Both analyses use the reference allele as this is more straightforward than using alternative alleles. Although you could expect binary alleles in individuals, in populations we often find that more than one alternative allele is present within a sample for any given SNP, and that the major alternative allele varies between samples. Grenedalf reports a reference allele count and an alternate allele count, but this is the common major allele across all samples not for each sample. PoPoolation2 reports what the common major allele is, but then reports the frequency of the major allele for each sample. For this reason, we choose to use the reference allele to be consistent across all samples.*

*For your interest, this is a plot of the F0 reference allele frequency of SNPs that were used in the model plotted using geom_hex (more SNPs under a hexagon = a higher count, one count per SNP). As you can see, most SNPs are found with a very high reference allele frequency:*

3. Have you attempted fine mapping using individual SNP CMH p-values or FST values? In the moxidectin analysis, did the authors observe a large number of individual SNPs with FST equal to 1? If so, this could make it difficult to further narrow down the selected locus using pool-seq data alone. In Fig. 6, a small number of individual SNPs show highly negative slope coefficients. Can the authors clarify whether these signals are biologically meaningful or likely to result from technical noise?

***RESPONSE:*** *We have not performed individual SNP Fst analysis due to the high number of SNPs present in the data. We did initially look at individual SNP p-values from the CMH test, however finding a realistic ‘cut-off’ proved impossible, with a huge number still significant even following either FDR or Bonferroni correction. We therefore did an analysis using the top 1% of SNPs by p-value that showed selection following treatment (negative log odds ratio) as this seemed a transparent and practical option.*

*Regarding your question about the SNPs from the AF model: there were only 12 SNPs and only one was both statistically significant and also strongly negative in more than one comparison. We have added information on these SNPs to a supplementary table, but cannot from their annotation information alone draw any conclusions as to their biological relevance.*

*Results and Discussion Section 5: “In total, 12 SNPs demonstrated a statistically significant, strongly negative slope coefficient of reduction (<-0.5) in the reference allele from F0 to F3 in selected lines. We have added information on all these SNPs to Supplementary Table S4.”*

4. The conclusion states that the findings of this study implicate pre-existence of “multiple” ivermectin and moxidectin resistance haplotypes in a drug-naïve population of H. contortus (line 704), but this claim lacks direct supporting evidence. With the available pool-seq data, it may be possible to investigate this by analyzing microhaplotypes (short stretches of DNA containing multiple linked SNPs). Several methods allow microhaplotype inference from Pool-seq data (e.g., PMID: 37923981). Analyzing the selected locus in this way could reveal whether multiple non-reference haplotypes were selected and whether the same haplotypes appear under both ivermectin and moxidectin selection.

***RESPONSE:*** *Thank you for suggesting this interesting analysis! Focusing on two single copy genes at either end of the locus, we obtained a selection of microhaplotypes along their lengths and plotted the allele frequencies, which we have included as Supplementary Figure 8. These demonstrate marked differences between control and selected lines. We have found that the same haplotypes appear to be selected by both ivermectin and moxidectin. However, moxidectin line 1, which showed maintenance of diversity in the previous analyses, has additional haplotypes which appear to be under selection by moxidectin. It also has a pattern for some microhaplotypes which is more similar to the ivermectin selected lines, where both a new haplotype is under selection and a control haplotype is maintained (Supplementary Figure 8).*

*We have amended the conclusion to read: “Our findings highlight the rapid selection for macrocyclic lactone resistance with subtherapeutic treatment and the pre-existence of resistance haplotypes in a drug-naïve population of H. contortus We demonstrate that ivermectin selected lines show emerging moxidectin resistance, underpinned by a shared genetic locus of resistance, with the same haplotypes under selection by both anthelmintics. However, striking differences in the resistance phenotype between ivermectin and moxidectin selected lines are apparent, which may relate to differences in the inheritance of resistance within this shared locus.”*

*We have also added the following to the results: “Finally, we looked at two single-copy genes located towards either end of the locus, calculating microhaplotype frequencies for these. We found that for each gene, a strong signal of selection was apparent for haplotypes that were distinct from those in control samples (Supplementary Figure 8). These haplotypes tended to be the same for both ivermectin and moxidectin selected lines, however the ivermectin selected lines tended to also retain moderate allele frequencies for the main control haplotypes, suggesting a dominant resistance mechanism under selection within the locus. In contrast, moxidectin lines 2 and 3 tended to show almost complete selection against the control haplotypes, suggesting a more recessive resistance mechanism. However, moxidectin line 1, which showed a higher diversity across the locus, often had distinct haplotypes appearing under selection. In addition, it often still showed similar allele frequencies for control haplotypes as the ivermectin lines.”*

5. The manuscript suggests that moxidectin resistance may be “more multigenic” than ivermectin resistance. However, the current findings are also fully consistent with a single gene model involving a recessive trait. The data do not directly support one interpretation over another. Please clarify that the proposed multigenic architecture is speculative and not clearly supported by the present data.

***RESPONSE:*** *We agree our interpretation is speculative until we have validated markers to follow in individual worms and the new microhaplotype analysis is consistent with moxidectin resistance being a recessive trait. We have moderated our interpretation throughout to be clear that moxidectin resistance appears to be recessive (based on previous work and our data) and while ML resistance could still be multigenic, this is speculative.*

*Abstract: “Finally, we speculate that key differences in the resistance phenotype between ivermectin and moxidectin selected lines relate to differences in the inheritance of resistance within this shared locus, with ivermectin resistance manifesting as dominant trait while moxidectin resistance appears to be recessive.”*

*Results and Discussion Section 4: “These findings could suggest a multigenic model of resistance, where a single variant (or multiple linked variants) confers resistance to a full dose of ivermectin and partial resistance to moxidectin, but where multiple discrete variants within the Chromosome V locus must be present in an individual worm for resistance to a full dose of moxidectin. They are also consistent with ivermectin resistance manifesting as a dominant trait while moxidectin resistance is recessive.”*

6. The authors could expand the discussion of how these findings apply to deworming practices in endemic areas. Additionally, limitations of the study should be stated more clearly. For example, it would be useful to comment on how well the experimental selection reflects real-world field dynamics, and whether these results are generalizable to O. volvulus.

***RESPONSE:*** *Thank you for this comment. We have added a paragraph stating limitations of the study (Results and Discussion Section 8) as follows ‘Limitations of the study include the use of pooled sequencing data throughout, so genotypes of susceptible and resistant individual worms could not be determined. The experimental selection design is relevant for subtherapeutic treatment of adult worms (underdosing) but does not replicate field dynamics, where incoming larvae will also be exposed to subtherapeutic concentrations of long acting anthelmintics (tail selection). Haemonchus contortus is a tractable parasitic nematode model, and findings from these experiments have direct relevance for anthelmintic treatment of closely related helminths of livestock, but it is currently unknown how generalisable the results are for more distant species, such as the filarial nematodes.’*

7. Please review the “Treatment Efficacy” column in Tables 3 and 4 to ensure correct number formatting.

***RESPONSE:*** *Done*

8. Given the broad readership of the journal, it would be helpful to briefly define or explain the following terms or acronyms at their first appearance, or where appropriate:

- Genetic basis
- Genetic architecture
- QTL
- FECR
- ED50

***RESPONSE:*** *All acronyms have been defined, and we have changed references to ‘genetic architecture’ to ‘inheritance’ based on the new microhaplotype analysis. Our preference is not to add an explanation for ‘genetic basis’ to avoid making the Author Summary overly long.*

9. Currently, only the BioProject ID is provided, individual sample accession numbers should be included in the supplementary information.

***RESPONSE:*** *Thank you - we apologise for overlooking this. Supplementary tables containing sample accessions and associated metadata are now available.*

10. Supplementary Fig. 2. There is a mismatch between the legend and the figure content. The legend refers to TBZ and TET, while the figure labels IVM and MOX.

***RESPONSE:*** *The legend refers to the drugs used in the assay while the figure titles refer to the drug-selected lines that were subjected to the assays. A statement has been added to the legend to clarify this.*

11. For Supplementary Fig. 7, the authors should clarify how read counts were normalized. Was normalization based on library size, and can the units be more descriptive to reflect the normalization used?

***RESPONSE:*** *The counts were normalised using ‘size factors’ as estimated by DESEQ2. For every gene, the size factor is calculated by measuring the geometric mean for that gene across all samples, dividing the count of the gene in each sample by the geometric mean (to provide a ratio), and taking the median of these ratios across all genes within a sample to generate a sample-specific size factor. The raw read count for each gene is normalised with this size factor. We have added a statement to the legend to specify that the read counts were normalised by size factor as per the DESEQ2 model.*

**Part II – Major Issues: Key Experiments Required for Acceptance**

Reviewer #1: Major comments
The idea of multi-drug resistance genes is repeated throughout the manuscript, and noted that none were observed in the current work. The authors mention detox pathways and P-glycoproteins as potential MDR mechanisms. On the first mention, the authors make the distinction that upregulation of these proteins “might” be expected. However, they seem to double down on the concept further in the manuscript. Although such pathways “could” be involved in MDR, no involvement has been validated at the molecular level. On the contrary, for the pathways that have been somewhat explored, such proteins are found to interact with specific classes, or in at least one case, of a single drug within the class. I suggest that the authors be more forthcoming that current data could argue against MDR-associated pathways and at a minimum acknowledge that either direction, broad or specific mechanisms, could be equally possible.

***RESPONSE:*** *Thank you for this comment – we agree that the role of detox pathways as MDR mechanisms is established in insecticide resistance but is speculative in helminths. We previously found upregulation of pgp-11 in both IVM and BZ selected populations of H. contortus, but this finding has not been validated at the molecular level. In the current study, there is no evidence of a role for detox/efflux pathways in resistance after selection with MLs exclusively, but it is at least possible that selection with BZs (for example) could induce upregulation of detox/efflux pathways that would confer a degree of resistance to the MLs – this has never been tested to our knowledge. We feel the reviewer’s comment is valid but already addressed in the following statement: ‘This is consistent with the expected independent mechanisms of resistance for each anthelmintic class (42), but also suggests that multi-drug resistance mechanisms, which we speculated could play a role in the initial stages of resistance evolution, are not relevant for emerging resistance to the macrocyclic lactones or at least are not observed with this experimental design.’*

Minor comments
I am generally aware of the history of the MHco3(ISE) isolate, but I think at a minimum, the information stating that it is fully susceptible should have relevant citations. Additionally, has the isolate been periodically phenotyped to confirm 100% susceptibility to common anthelmintics? Lastly, has the current stock of the isolate been confirmed to be molecularly pure H. contortus?

***RESPONSE:*** *Thank you for this comment, which is something we have also given thought to. The MHco3(ISE) isolate is derived from the SE isolate, which was originally isolated in South Africa and has been maintained at the Moredun since the late 1950s – this predates all use of macrocyclic lactones, which were first released in the 1980s. Citations for this have been added to the methods. The high efficacy of extremely low doses of ivermectin and moxidectin in the first generation, and the low ED50s of the control lines in the larval development assay, are both consistent with full susceptibility. We are not aware of a more sensitive test of susceptibility that we could perform and whether any natural population would be 100% susceptible at subtherapeutic doses as low as in the current study is not known. In the L3 whole genome sequencing data, 99.3% (SD 0.2%) of ITS2 reads were “Haemonchus genus” and manual picking of adult worms (all Haemonchus based on morphology) confirm that the current stock is molecularly pure H. contortus.*

Line 95-99: There has not been confirmed validation of a gene associated with IVM/MOX resistance, and cky-1 (found through BSA) has also not been validated as the causal gene. My question is what evidence supports that the animals in the cited work were truly heterozygous vs homozygotes at relevant loci and how does this change the interpretation of the concept being presented?

***RESPONSE:*** *All data in this study are from pooled populations, so it is not possible to determine individual worm genotypes other than where variants are at fixation in a population. We have added a statement to this effect in the new paragraph on limitations. We are working to identify the causal variant(s) which will allow us to determine whether resistant worms are heterozygous or homozygous at relevant loci with single worm sequencing.*

Line 180: Were the bags simply sealed in ziplock bags as opposed to vacuum sealing? How long were the plates stored? Both could impact the activity of the drug if the plates were stored for extended lengths of time or in a way that could allow some desiccation.

***RESPONSE:*** *All pre-treatment drug plates were prepared at the same time to minimise batch effects and sealed, without vacuum, for short-term storage at 4 °C. The assays were run 1-3 days later (animal work was staggered over three days with one control, one ivermectin-selected and one moxidectin-selected line/animal per day). The same protocol was followed for the post-treatment drug plates. For assays with ivermectin aglycone, there is some indication of increasing ED50 across the three days in the replicated controls, however, this is not observed in the replicated selected lines, so any effect does not seem to be large, although it is certainly something we will consider in future work. If there was to be a difference in drug activity over time, this would be corrected in the resistance ratios, which were calculated using the same-day controls.*

Line 187-191: Were the plate concentrations of drugs made with knowledge that 40 µL of additional volume would be added? With the addition of the embryos and nutritive media, the concentration of drugs in the well would have significantly decreased after absorption into the agar, impacting the results. If concentrations were not adjusted for this dilution, actual exposure concentrations should be calculated and the data should be interpreted given the actual concentrations.

***RESPONSE:*** *The larval development assay plates were made using the standard protocol for DrenchRite plates; these have the same additional volumes of eggs in water and nutritive media, and the drug concentrations in the agar at the start of the experiment are reported. While the reviewer is correct that after the addition of eggs and nutritive media, the drug concentration in the well will be lower than in the agar at the start of the experiment, we would like our results to be consistent and directly comparable to other published work using the same assay, so our preference is to report the concentration in the agar as per standard practice.*

Line 233: It appears that you discuss how indels were called in the sequences, but I do not see how SNVs were called in the methods, although below they are discussed as part of the analysis for variants. Please add these details.

***RESPONSE:*** *Thank you for this question. The indel detection described on lines 236-240 is actually a processing step to help remove potential error base alignments (false SNVs) introduced due to incorrect alignment around an indel and is part of the recommended pre-processing in the PoPoolation2 manual. After identifying an indel, up to 5 basepairs around it are removed from the data, reducing errors. All SNVs and indels are present within the mpileup/sync files (essentially just counts of each base, indels and Ns for each genomic site) and are detected and filtered or kept by Grenedalf and PoPoolation2 used to analyse them based on the filters set by the user.*

Line 245: To only cover 31.7% of the total bases in the assembly leads me to believe that there are elements of diversity that would be missed in most of the genome and not factored into the current analysis. Was there a reason that only one third of the genome was considered instead of doing further sequencing?

***RESPONSE:*** *Yes, you’re right - subsampling the genome did remove certain data from consideration. However, for the Fst and diversity analyses, variation in depth would influence the statistical outcome, and so we decided to subsample the reads to produce an even coverage for these analyses (the subsampled sync file). Due to uneven coverage across the genome and between samples, only a third of all genomic positions were retained in the subsampled file as having read depth of 57X or greater in all samples. Although we discarded 60% of the genome for these analyses, the remaining SNP density was still sufficient to identify regions under selection.*

*For analyses which relied on the SNPs themselves (e.g. the CMH and allele frequency model), these were performed with all data (‘complete mpileup file’) i.e. without first subsampling. To account for variable depth, the allele frequency was calculated using the reference allele count and the total depth as calculated from the mpileup file. Increasing sequencing depth by additional sequencing of samples would still produce uneven depth due to sequencing bias, and diverse reads which fail to align to the genome, or which align in multiple locations (MAPQ=0) - the genome contains a lot of repetitive sequence.*

Line 294: I assume the window on chromosome V was chosen due to the previous data indicating an interval containing cky-1 as a candidate gene for ML resistance, but the previous work is not mentioned to justify why this window was chosen. I see that the first mention of this work is at line 465, but please discuss prior to this point the previous work so that this window is justified in context.

***RESPONSE:*** *We have added the following statement to the methods “(identified as the major region under drug selection, based on the Fst and CMH analyses above)”.*

Line 422-425: As a Discussion point, consider briefly presenting the implications of how quickly ivermectin resistance would become highly moxidectin-resistant in a field population.

***RESPONSE:*** *This is a very interesting question, but on the basis of this study we cannot speculate on the speed of selection for high level moxidectin resistance in the field once ivermectin resistance has arisen, other than to suspect it would be faster than in an ivermectin-susceptible population.*

Figure 2: If axis labels are going to be used for all the plots in the figure, please ensure that the labels are cleanly shown for all plots, as currently the genomic position seems to be specific for plot D, but applies to all plots. I also suggest adding more space between your lettering for each plot and the y-axis labels.

***RESPONSE:*** *Done*

Figure 3 and other figures: This may be an effect of the upload for the draft, but please ensure all figures are of publication quality. Currently, some figures are grainy/pixelated in the draft. I also suggest adding more space between your lettering for each plot and the y-axis labels, and this issue is present in multiple figures.

***RESPONSE:*** *Done*

Line 644-645: Are any of the DEGs known to be involved in drug response or could make sense in the context of ML resistance?

***RESPONSE:*** *Please see response to related comment above.*

Table 6. I suggest that, where possible, specific gene names (or C. elegans orthologs) be listed. The HCON_**** nomenclature is not informative without actually having to search for each gene. Additionally, the HCON_00158160 gene ID does not appear in a search of the genome. Please confirm that the listed IDs are accurate.

***RESPONSE:*** *Thank you for this comment. Our preference is to use the H. contortus gene IDs to avoid ambiguity, but we have added the Interpro domain where present and name of the closest C. elegans homologue where one exists (Supplementary Table 5). We believe the gene IDs are correct and gene HCON_00158160 is in the current annotation of the genome, please see* [*https://parasite.wormbase.org/Multi/Search/Results?species=all;idx=;q=HCON_00158160;site=ensemblunit*](https://parasite.wormbase.org/Multi/Search/Results?species=all;idx=;q=HCON_00158160;site=ensemblunit)

Specific comments
Line 145-149: I suggest separating the very long sentence about dosing into two separate doses. Currently, it comes off somewhat confusing with the “then ivermectin… and .008 mg/kg…”

***RESPONSE:*** *Done*

Line 186: Please cite a reference for “standard procedures”.

***RESPONSE:*** *Done*

Line 405: The use of recapitulated for something that has not previously been validated seems like awkward language. I suggest simply saying that MDR mechanisms were not observed.

***RESPONSE:*** *Done*

Reviewer #2: None required

Reviewer #3: (No Response)

**Part III – Minor Issues: Editorial and Data Presentation Modifications**

Reviewer #1: (No Response)

Reviewer #2: The manuscript was generally well written but at times the use of 'three third generation' and similar phrases a bit clumsy. As the narrative continues there is use of F0, F1, F2, F3 which was much easier to digest - perhaps these could be introduced earlier e. g 3 x F3 rather than 'three third generation'.

***RESPONSE:*** *Thank you for this suggestion. We agree the phrasing is longwinded but given we are using the L3 offspring as a proxy for selection in the adult worms, we feel it is clearer and more intuitive to refer to the generation of selection e.g. third generation of selection in adults, pre-treatment L3 and post-treatment L3 (rather than F3 adults post treatment, F4 L3 pre-treatment of F3 adults and F4 L3 post-treatment of F3 adults). However, we have edited where possible to make more concise e.g. ‘the three third generation’ to ‘all third generation’.*

The experimental work in animals is well explained but is complex, I think an inclusion of an workflow (as supplementary?) or infographic showing these experiments would help with understanding. Given the extensive and well conducted molecular and genomic work is carried out on these F0-F4 populations it is crucial that this is well explained and I think a workflow/infographic of the sheep experiments would really help.

***RESPONSE:*** *Done*

The larval assays indicated that moxidectin resistance demonstrate side resistance to ivermectin, it is a shame that there is not in vivo data also showing this, for example treatment of IVM-selected F3 with MOX in vivo, and vice versa. These experiments were not done, but given the work involved I am not proposing they must be, rather just a mention of the that this might have been an expected outcome had they been done.

***RESPONSE:*** *Thank you for this comment. As suggested by the reviewer, we were able to treat the IVM-selected F3 lines with MOX in vivo and show a reduced efficacy relative to the control F3 (Table 3). We did not however test the IVM resistance of the MOX-selected F3 lines in vivo, largely because the phenomenon of side resistance in this direction is already recognised in the field. Based on the ED50 values from the LDAs in this project, and extrapolating from previous LDAs of ivermectin resistant field samples (already described in the results and discussion), we would expect the MOX-selected lines to be IVM resistant, but we agree this would need to be tested in vivo to confirm.*

There were a couple of results that were intriguing that could perhaps be discussed a little further:
1. The apparent rapid selection of resistance seen by the FECRT data by both MOX and IVM following just one treatment with a subtherapeutic dose is somewhat surprising. How does this observation align with the development of resistance in naturally infected sheep and cattle? For example over what time frame has resistance developed in Haemonchus populations. I am just wondering, despite the ISE isolate still displaying substantial genetic diversity if these pre-existing alleles are at higher frequency in the F0 population than in WT populations of Haemonchus. Perhaps a line to discus this?

***RESPONSE:*** *We agree the change in phenotype after a single dose is striking, but not without precedent. Previous selection experiments by Coles et al (2005) also found rapid selection for full ivermectin resistance in 3 generations using two distinct Haemonchus isolates (one from the UK, one from the US). While the method was different in that study (multiple doses given to each generation of adult worms), by the second generation, the adults survived a higher dose of ivermectin than used in our study. The findings are also consistent with the development of ivermectin resistance in the field, at least under conditions of presumed low refugia: one of the first reports of resistance was in two South African populations of H. contortus after a history of only 3 treatments each (van Wyk et al, 1989). To provide context to our results, we have added the following:*

*Results and Discussion Section 7: “Resistance to ivermectin can develop very rapidly in H. contortus; early reports of resistance in the field involved two populations of H. contortus with a history of only three ivermectin treatments (Van Wyk et al., 1989)”*

2. Given there are the two sexes and known differences in expression patterns between males and females, what attempts were made with the 5000 L3 and the batches of 200L3 to ensure they represented a mix/balance of the two sexes. Is there any data, or easily accessible method, of determining the species mix of the larvae?

***RESPONSE:*** *There is no easily accessible way to determine the sexes of the batches of L3, but we performed RNA-seq analysis on both male and female adults separately to ensure any sex-specific differences in gene expression could be detected.* *We are confident that pure populations of H. contortus were used in the study; please see our response to similar comment from reviewer 1.*

Reviewer #3: (No Response)
